# Mitochondrial dynamics in parasitic protists

**DOI:** 10.1371/journal.ppat.1008008

**Published:** 2019-11-21

**Authors:** Luboš Voleman, Pavel Doležal

**Affiliations:** Department of Parasitology, Faculty of Science, Charles University, BIOCEV, Prague, Czech Republic; Boston College, UNITED STATES

## Abstract

The shape and number of mitochondria respond to the metabolic needs during the cell cycle of the eukaryotic cell. In the best-studied model systems of animals and fungi, the cells contain many mitochondria, each carrying its own nucleoid. The organelles, however, mostly exist as a dynamic network, which undergoes constant cycles of division and fusion. These mitochondrial dynamics are driven by intricate protein machineries centered around dynamin-related proteins (DRPs). Here, we review recent advances on the dynamics of mitochondria and mitochondrion-related organelles (MROs) of parasitic protists. In contrast to animals and fungi, many parasitic protists from groups of Apicomplexa or Kinetoplastida carry only a single mitochondrion with a single nucleoid. In these groups, mitochondrial division is strictly coupled to the cell cycle, and the morphology of the organelle responds to the cell differentiation during the parasite life cycle. On the other hand, anaerobic parasitic protists such as *Giardia*, *Entamoeba*, and *Trichomonas* contain multiple MROs that have lost their organellar genomes. We discuss the function of DRPs, the occurrence of mitochondrial fusion, and mitophagy in the parasitic protists from the perspective of eukaryote evolution.

## Mitochondrial dynamics in model cellular system of yeast and humans

**Fig 1:**
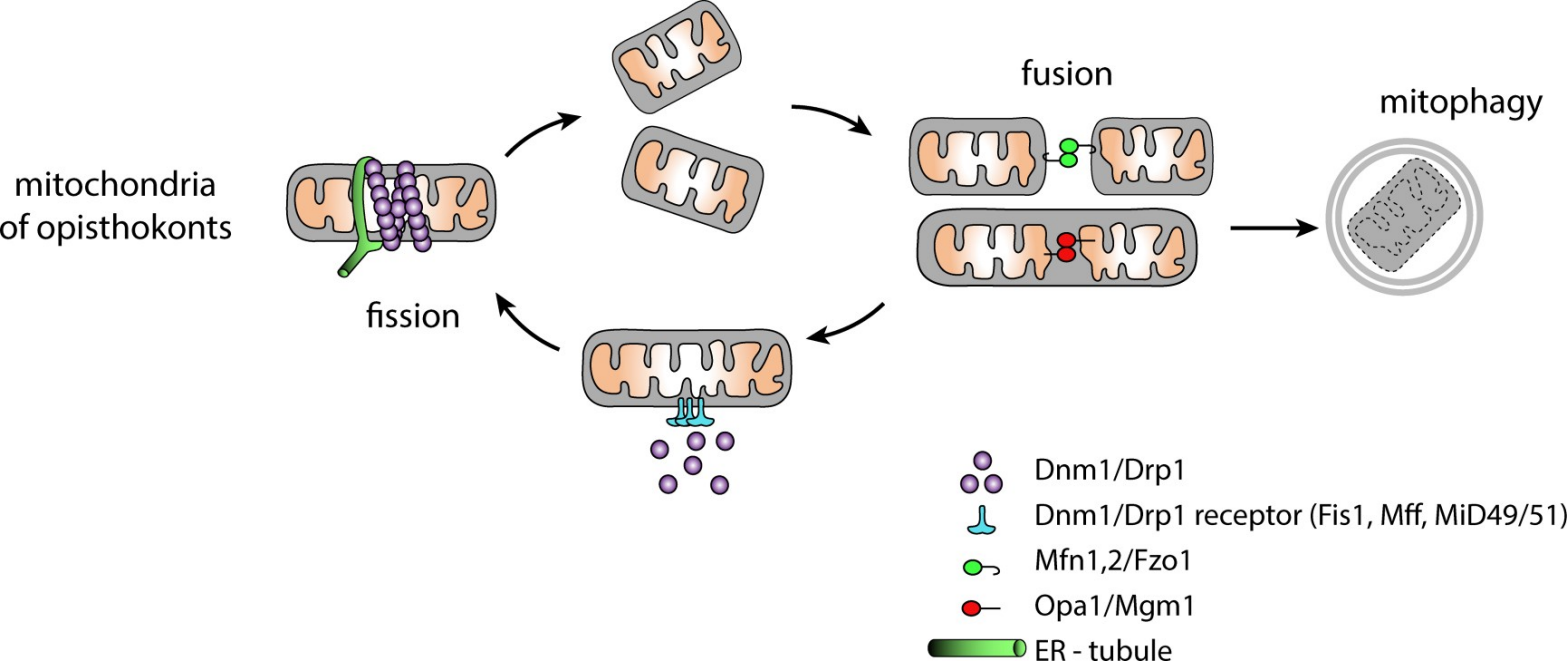
Mitochondrial dynamics. Mitochondria of animals and fungi undergo constant cycles of opposing fission and fusion events. For division, the molecules of DRP are recruited to the mitochondrial surface by DRP receptors. The sites of membrane fission often coincide with the ER-mitochondria connections. Membrane-anchored DRPs specific for the outer (Mfn1,2/Fzo1) and the inner (Opa1/Mgm1) mitochondrial membrane mediate the fusion. Defective mitochondria are removed from the cycle by mitophagy. DRP, dynamin-related proteins; ER, endoplasmic reticulum.

Mitochondria are semiautonomous organelles cordoned by two membranes that are not contiguous with the endomembrane system of the eukaryotic cell [1]. As studied in model cellular systems of yeast and human cells (representatives of the Opisthokonta supergroup of eukaryotes), the mitochondria constantly divide and fuse during the cell cycle. Discrete organelles are thus in a dynamic balance with the continuous and reticulate network [2]. This dynamic responds to the metabolic state of the cell, supporting the idea that mitochondrial fusion functionally complements defective organelles via the exchange of proteins, lipids, and DNA [3,4]. The organellar dynamics require precise control over the membrane fission and fusion events, which are mediated by dynamin-related proteins (DRPs) [5] (Fig 1). Fission relies on the assembly of a Drp1/Dnm1 helical oligomer (mammalian/yeast nomenclature) on the mitochondrial membrane [6], which constricts the membrane upon GTP hydrolysis [7]. The soluble cytosolic Drp1 must, however, be recruited to the mitochondrial surface by sets of receptors that are, except for Fis1, different for human and yeast systems (Mff, MiD49, MiD51, and Fis1 for human [7,8] and Mdv1, Caf4 and Fis1 [9,10] for yeast mitochondria).

In addition to the protein machinery, mitochondrial division is assisted by the tubules of the endoplasmic reticulum (ER), which seem to wrap around the constriction sites [11,12]. In yeast, these ER-mitochondria hotspots are defined by the molecular tethering complex known as ER-mitochondria encounter structure (ERMES) [13].

The fusion of mitochondria requires two membrane-anchored DRPs specific for the two mitochondrial membranes, mitofusins (Mfn1, Mfn2/Fzo1) [14,15] in the outer membrane and Opa1/Mgm1 in the inner membrane, respectively. In yeast, the fusion of the two membranes is coordinated by Ugo1, which interacts with both Fzo1 and Mgm1 [16], and a homologous role has been proposed for mammalian SLC25A46 [17].

Finally, defective mitochondria are removed from division–fusion cycles by mitophagy, a specific autophagy pathway [18] that leads to lysosomal degradation of those organelles.

The molecular machineries and the regulatory pathways controlling mitochondrial dynamics have been dissected at a molecular level in selected opisthokont cellular models. However, the aspects of mitochondrial dynamics outside the opisthokonts remain a highly unexplored field. In parasitic protists, two opposing factors have affected mitochondrial dynamics. Parasitism has streamlined the overall cellular structure including mitochondria, whereas complex life cycles have often resulted in diverse specialized parasite stages. Here, we review current knowledge on the mitochondrial dynamics of medically important protist parasites, namely, *Plasmodium* spp., *Toxoplasma gondii*, *Trypanosoma brucei*, *Trichomonas vaginalis*, *Giardia intestinalis*, and *Entamoeba histolytica*.

### *Plasmodium* spp.

**Fig 2:**
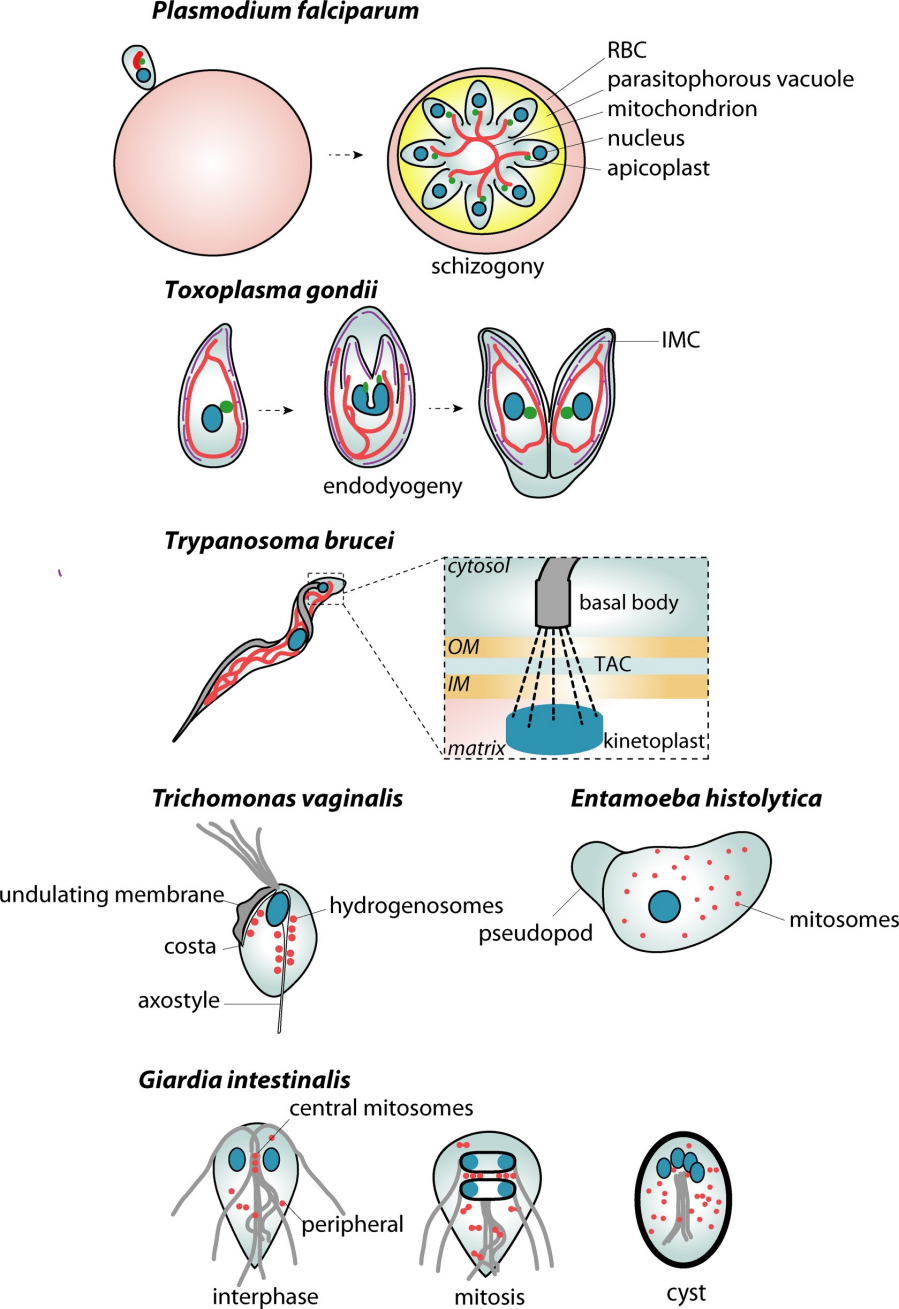
Key aspects of mitochondrial dynamics in parasitic protists. Single mitochondrion of *P*. *falciparum* in synchrony with the specific cell division known as schizogony. The elongated mitochondrion branches and segregates into emerging daughter cells at the very end of the cell division. Throughout most of the cell cycle, the mitochondrion maintains the association with the apicoplast. Similarly, two daughter cells of *T*. *gondii* develop within the mother cell by a process known as endodyogeny. Here, the mitochondrion is also in contact with the IMC—a membranous compartment underneath the cytoplasmic membrane specific to Alveolata. In *T*. *brucei*, the mitochondrial genome is arranged into a kinetoplast disc, which is physically connected to the basal body of the flagellum by a TAC. The division of the single parasite mitochondrion is thus fully coordinated with the maturation of a new flagellum and the segregation of two daughter kinetoplasts. MROs of anaerobic parasitic protists are always present in higher numbers. In *T*. *vaginalis*, hydrogenosomes associate with the main cytoskeletal structures, the axostyle and costa. In *G*. *intestinalis*, central and peripheral mitosomes can be distinguished. The first are proposed to be in contact with the axonemes of flagella. Both populations divide during mitosis, which is also part of the encystation process. The mature cyst thus contains a doubled set of nuclei and mitosomes. In *E*. *histolytica*, hundreds of mitosomes are distributed all over the cell. IM, inner mitochondrial membrane; IMC, inner membrane complex; OM, outer mitochondrial membrane; MRO, mitochondrion-related organelle; RBC, red blood cell; TAC, tripartite attachment complex.

*Plasmodium* parasites belong to phylum Apicomplexa, most of which are obligatory intracellular parasites with a highly specialized cellular structure for cell invasion, the apical complex [19]. Multiple *Plasmodium* species cause malaria in humans and other vertebrates, and these parasites go through a series of morphological transformations during their life cycle, spanning the intermediate vertebrate host and the definitive host, the mosquito [20]. In the vertebrate host, the parasite asexually reproduces first within hepatocytes, dividing to form merozoites that burst from the hepatocyte to invade erythrocytes [20]. There is a single mitochondrion in each *Plasmodium* cell, the energy metabolism of which is suppressed at the intraerythrocytic stage [21] as manifested by the loss of cristae [22]. Within the erythrocyte, the parasite undergoes the unusual process of schizogony, a series of nuclear divisions without cytokinesis, which gives rise to multinucleated schizonts. As the parasite grows, the mitochondrion branches massively and segregates into emerging daughter cells [23] (Fig 2). The mitochondrion associates with the apicoplast [23–25], a secondary plastid harbored by most apicomplexans [26], and this association becomes more prominent during schizogony, when both organelles divide. The actual mitochondrial division always occurs after the apicoplast has been divided (Fig 2). The coordinated division and the mutual contacts of both organelles may represent a mechanism ensuring that every daughter cell always receives one copy of each organelle [23,27]. During the initial schizogony in the liver, the divisions occur on a much larger scale generating thousands of nuclei. Nevertheless, the overall sequence of division events is analogous to the erythrocytic stage [28,29]. Here, however, the mitochondrion forms enormous branched structures intertwined with the apicoplast [28], and the association between the apicoplast and mitochondrion is not seen during schizogony until the apicoplast starts to divide [28,29]. Before the transmission to mosquito, the parasite undergoes differentiation into sexual stages (gametocytogenesis), during which the mitochondrion’s morphology changes dramatically [30]. Within the erythrocyte, the precursors of female and male gametes are formed (macrogametocyte and microgametocyte, respectively). The mitochondrion of the macrogametocyte elongates and branches, eventually forming a cluster around the apicoplast, which itself does not change [30]. Upon transmission to the mosquito, gametocytes rapidly differentiate to mature gametes, and a macrogamete and a microgamete fuse to produce a zygote that later develops into the infectious sporozoites [30]. The fusion of the gametes does not involve the fusion of their mitochondria, as only the female’s mitochondrion is retained [31]. In fact, mitochondrial fusion has not been observed in *Plasmodium* [28]. This corresponds to the lack of mitofusins in the genome sequences of *Plasmodium* species (Table 1), although an alternate molecular machinery may be in charge. Despite the observed massive synchronized mitochondrial division, there is no information on the involvement of the only two dynamin orthologues (Dyn1 and Dyn2) identified in *Plasmodium* genome [32–35]. While Dyn1 has been suggested to participate in the hemoglobin uptake, Dyn2 partially localizes to the endomembrane system and the apicoplast [32]. Thus, the molecular fission machinery of *Plasmodium* mitochondrion remains unknown.

**Table 1:** Distribution of components involved in mitochondrial division and fusion in parasitic protists.

	Drp1/ Dnm1	Drp1/Dnm1 receptors			Fusion	Mitofusin	ERMES	Mitophagy
Species		Fis1	Mff MiD51 MiD49	Caf4 Mdv1				
***Plasmodium falciparum***	32–34	35						
***T*. *gondii***	34,40,42	42						45,46
***Cryptosporidium sp*.**	98							
***T*. *brucei***	56,58,89	56,58,59			63?	60?	83,99,100^#^	
***Leishmania sp*.**	58						83	101
***T*. *vaginalis***	74							102
***G*. *intestinalis***	78,81,82							
***E*. *histolytica***	87,88				89?			

White fields depict the presence of the orthologous protein; references to corresponding publications are shown. The phenotypic observation of mitochondrial fusion or mitophagy is also marked. Question mark depicts the indirect evidence.

^#^Homologues of ERMES components present but neither form a complex nor participate in the ER-mitochondrion contact sites.

**Abbreviation:** ERMES, ER-mitochondria encounter structure

### Toxoplasma gondii

*T*. *gondii* is an apicomplexan parasite that infects many warm-blooded animals (including humans) as possible intermediate hosts. The definitive hosts are felids [36]. The mitochondrion of *T*. *gondii* has been described predominantly in a rapidly proliferating life stage called tachyzoite. The single mitochondrion of *T*. *gondii* clusters at the anterior part of the cell with branches spanning towards its posterior part, forming a lasso shape [37]. In a pattern analogous to *Plasmodium*, the mitochondrion of *Toxoplasma* undergoes striking extension and branching during the cellular division process known as endodyogeny (Fig 2). In contrast to schizogony of *Plasmodium*, only two daughter cells are formed within the mother cell [38]. The mitochondrial extensions continue to grow, ultimately surrounding the growing daughter cells. Interestingly, mitochondrial branches enter the developing daughter cells at the last possible moment of cell division, migrating along the cytoskeletal scaffolding. A small amount of mitochondrial compartment is left behind in the residual body [39].

The mechanism underlying division of the two emerging mitochondria has been partially uncovered only very recently. There are three DRPs encoded in the *T*. *gondii* genome. *Tg*DrpA and *Tg*DrpB are involved in the biogenesis of apicoplast and micronemes/rhoptries, respectively [34,40], and *Tg*DrpC was shown to associate with multiple compartments, including mitochondria [41,42]. *Tg*DrpC is specific to apicomplexans and closely related organisms [34] and is a highly divergent DRP as it contains only the N-terminal GTPase domain and lacks both the central and GED (GTPase effector) domains [41,42]. This indicates that the protein is a lineage-specific protein invention. *Tg*DrpC forms puncta in the cytosol, some of which are in close proximity to mitochondrial membrane. By modifying the amount and primary structure of *Tg*DrpC, prolonged mitochondria and other defects in mitochondrial morphology could be observed, suggesting its specific involvement in mitochondrial division [42]. However, *Tg*DrpC seems to have rather pleiotropic functions, given that the morphology of other compartments like Golgi, apicoplast, and the inner membrane complex (IMC) also changed in the affected cells [41].

The genome of *T*. *gondii* also encodes a homologue of the adaptor protein, Fis1. In contrast to yeast Fis1 but similar to the human homologue, knockdown of *Tg*Fis1 did not affect mitochondrial morphology or parasite proliferation [42]. Thus, the actual role of Fis1 in the protist remains unknown. So far, no fusion between mitochondrial tubules has been directly observed in *T*. *gondii* (Table 1), yet the formation of the lasso structures indicates at least a single fusion of the mitochondrial tubules.

The mitochondrion of *T*. *gondii* was also reported to associate with the apicoplast during parasite division, but this association is not maintained throughout the whole process [39]. The mitochondrion of tachyzoites was instead shown to associate with the IMC, a unique arrangement of flattened membranes found directly below the plasma membrane of apicomplexans [43,44] (Fig 2). Mitochondrial morphology also relates to the egress of the parasite from the host cell, during which the lasso-shaped mitochondrion concentrates from the cell periphery into so-called "condensed" and "sperm-like" morphologies [44]. These structures are preserved until the induction of gliding movement and the invasion into the new host cell. Consistently, collapsed mitochondria of invading parasites were shown to re-expand and re-establish a lasso shape via the sperm-like intermediate, and only parasites that harbor lasso-shaped mitochondrion are able to divide within the host cell [44]. Interestingly, the integrity of single *T*. *gondii* mitochondria was found to be somehow controlled by autophagy [45,46]. The deletion of Atg3, an essential autophagy factor, caused specific morphological defects to the mitochondrion [46]. On the other hand, upon starvation, during which the formation of the autophagosomes is induced, the mitochondrion became irreversibly fragmented. This fragmentation could be blocked by an autophagy inhibitor, 3‐methyl adenine [45]. Thus, the exact functional connection between autophagy and mitochondrial biogenesis/dynamics must be studied further.

### Trypanosoma brucei

The single mitochondrion of *T*. *brucei*, a causative agent of African trypanosomiasis [47], undergoes dramatic metabolic changes during the parasite life cycle [48]. These changes are reflected by the shape and metabolic capacity of the mitochondrion. In the tsetse fly vector, proline is the main energy source, and mitochondrion catalyzes its oxidation via the Krebs cycle [48]. Accordingly, the mitochondrion develops a highly branched network with distinct inner membrane cristae. On the other hand, the bloodstream form (BSF) of the parasite takes advantage of glucose abundance, and its mitochondrion becomes functionally repressed and acristate [48]. The recently discovered adipose tissue form of the parasite also revealed reduced mitochondrial volume [49]. The machinery responsible for the direct morphological transformation of the organelle is not understood. However, as shown in BSF, after cytokinesis the mitochondrial network gradually develops by branching of a single mitochondrial tube [50]. The most striking character of *T*. *brucei* mitochondrion is the presence of the kinetoplast, a highly ordered mitochondrial DNA [51]. The kinetoplast is physically connected to a basal body of the single flagellum via the so-called tripartite attachment complex (TAC) [52]. The division of the mitochondrion is thus fully coordinated with the maturation of a new flagellum and the segregation of two daughter kinetoplasts [53–55]. Importantly, defects in mitochondrial division result in cell cycle arrest [56], and the division of the organelle may thus serve as a cell cycle checkpoint [57]. *T*. *brucei* encodes orthologues of two mitochondrial division proteins, Drp1 and Fis1 [56,58] (Table 1). The dynamin-like protein, TbDLP, is present as two highly similar paralogues [59]. These are localized not only in the mitochondrion but also in the flagellar pocket compartment, where endocytosis occurs [56]. The ablation of TbDLP thus leads to impaired mitochondrial division as well as endocytosis [56,58]. So far, mitochondrial fusion has not been directly reported in trypanosomes. However, a candidate mitofusin (TbMNFL) homologue was identified in trypanosomes [60]. Silencing the expression of TbMNFL resulted in a mitochondrial fenestration phenotype, which is rather reminiscent of the fission defect in yeast mitochondria [60,61]. Whether the protein is dedicated to mitochondrial fusion requires further examination. Moreover, mitochondrial fusion may be limited to a specific life cycle stage of *T*. *brucei*, as haploid, gamete-like *T*. *brucei* cells that differentiate in tsetse flies undergo cellular fusion [62]. However, it was shown that mitochondria of BSF of *T*. *brucei* artificially fragmented by expression of the human proapoptotic protein Bax are capable of refusion into one single organelle after halting Bax expression [63]. Recently, frequent fusion events between mitochondrial tubules were reported in a related kinetoplastid organism, *Crithidia fasciculata* [64], which suggests that trypanosome mitochondrion may also fuse under physiological conditions.

### Trichomonas vaginalis

*T*. *vaginalis* is a causative agent of trichomoniasis, the most common sexually-transmitted disease of nonviral origin [65]. Instead of mitochondria, *T*. *vaginalis* contains rounded organelles about 1–2 μm in diameter called hydrogenosomes, which belong to a large group of so-called mitochondrion-related organelles (MROs) [66]. Hydrogenosomes are devoid of DNA and cristae and produce ATP by substrate-level phosphorylation with concomitant production of hydrogen. However, substantial biochemical and phylogenetic evidence has demonstrated that hydrogenosomes are anaerobic adaptations of mitochondria, and the general blueprint of organelle biogenesis is shared between both types of the organelles [67,68]. Over a hundred hydrogenosomes are localized along the axostyle and costa, unique cytoskeletal structures, which may consume hydrogenosome-generated ATP [69]. Morphological studies described several types of hydrogenosomal division and their association with the ER [70,71]. However, mainly due to the lack of live imaging data [72,73], it is possible that all division types are merely different morphological manifestations of a single division process, or perhaps some of them represent distinct hydrogenosome-related pathways. There are eight DRPs in the genome of *T*. *vaginalis*. One of them was localized to the hydrogenosomes, and the expression of the dominant-negative form of the protein resulted into the presence of larger and fewer organelles [74]. This was likely a consequence of impaired hydrogenosomal division, hence strongly suggesting the role of this single DRP in the process [74]. So far, fusion between hydrogenosomes has not been observed, nor have mitofusin orthologues been identified in *T*. *vaginalis* genome.

### Giardia intestinalis

*Giardia intestinalis* possesses some of the most reduced MROs, known as mitosomes [75]. About 40 organelles 50–200 nm in size harbor single metabolic pathway of the iron–sulfur cluster biosynthesis [76–78]. Mitosomes do not generate ATP but are still bounded by two membranes [75].

Two main populations of mitosomes are recognized in *G*. *intestinalis*: central mitosomes (CMs)—located between the two *Giardia* nuclei, where basal bodies of eight flagella are also localized—and the peripheral mitosomes (PMs), distributed in the rest of the cell [79,80]. Regarding the conserved position of CMs, it was proposed that CMs might be connected to the basal bodies and that their division could be coordinated with the parasite cell cycle [79].

There is only a single DRP in *G*. *intestinalis* (GlDRP), and its involvement in mitosomal dynamics remains unclear [78,81]. The main localization of the protein at the cytoplasmic membrane indicated its role in the scission of the endocytic vesicles, whereas the dominant-negative form of GlDRP interfered with the differentiation of the parasite into the cyst stage [81,82]. The mutant form of the protein also colocalized with the mitosomes, where it possibly interfered with mitosomal division [78]. However, a different study could not confirm the role of GlDRP in mitosomal division [81], and more experiments will be needed to clarify the involvement of this protein.

Interestingly, mitosomal dynamics are in synchrony with the cell cycle as both CMs and PMs divide only during mitosis [81]. This includes encystation of the parasite, during which the cell undergoes another round of mitosis without cytokinesis, and the mitosome number doubles accordingly. No fusion between mitosomes was observed in *G*. *intestinalis* even upon several hours of long observations [73,78,81], which is supported by the absence of mitofusins in the genome sequence. However, despite the obvious lack of the ERMES complex components in *G*. *intestinalis* [83], mitosomes remain in very close contact with the ER throughout the parasite cell cycle and life cycle. Moreover, the contact sites between mitosomes and the ER are enriched for long-chain acyl-CoA synthetase, which indicates synthesis and/or transfer of lipids between these two organelles [81].

### Entamoeba histolytica

From a functional standpoint, the mitosomes of *E*. *histolytica* represent a uniquely redesigned mitochondrial compartment [84]. They harbor just a single pathway of sulfate activation, components of which have been obtained by lateral gene transfer from different eukaryotic and bacterial sources [85] and which is specifically needed during the encystation of the parasite [86].

Four DRPs have been identified in the *E*. *histolytica* genome [87,88]. Of these four, two proteins (EhDrpA and EhDrpB) were shown to localize to *Entamoeba* mitosomes [88]. They contain all necessary functional domains, and, importantly, the expression of corresponding dominant-negative forms caused dramatic elongation of the organelles, suggesting their specific involvement in the mitosomal division. Interestingly, both DRPs are functionally interdependent. They interact together and their function is not mutually substitutable [88]. These data suggest that *E*. *histolytica* requires a formation of heterooligomeric DRP complex for the mitosomal division, so far unseen in other cellular systems.

Direct fusion of *E*. *histolytica* mitosomes has not been observed so far. However, a recent study indicated fusion between endogenous and microinjected mitosomes in manipulated *E*. *histolytica* cells [89]. However, the molecular machinery and the physiological relevance of the observation remain to be tested.

## Ancestral or acquired similarities?

**Fig 3:**
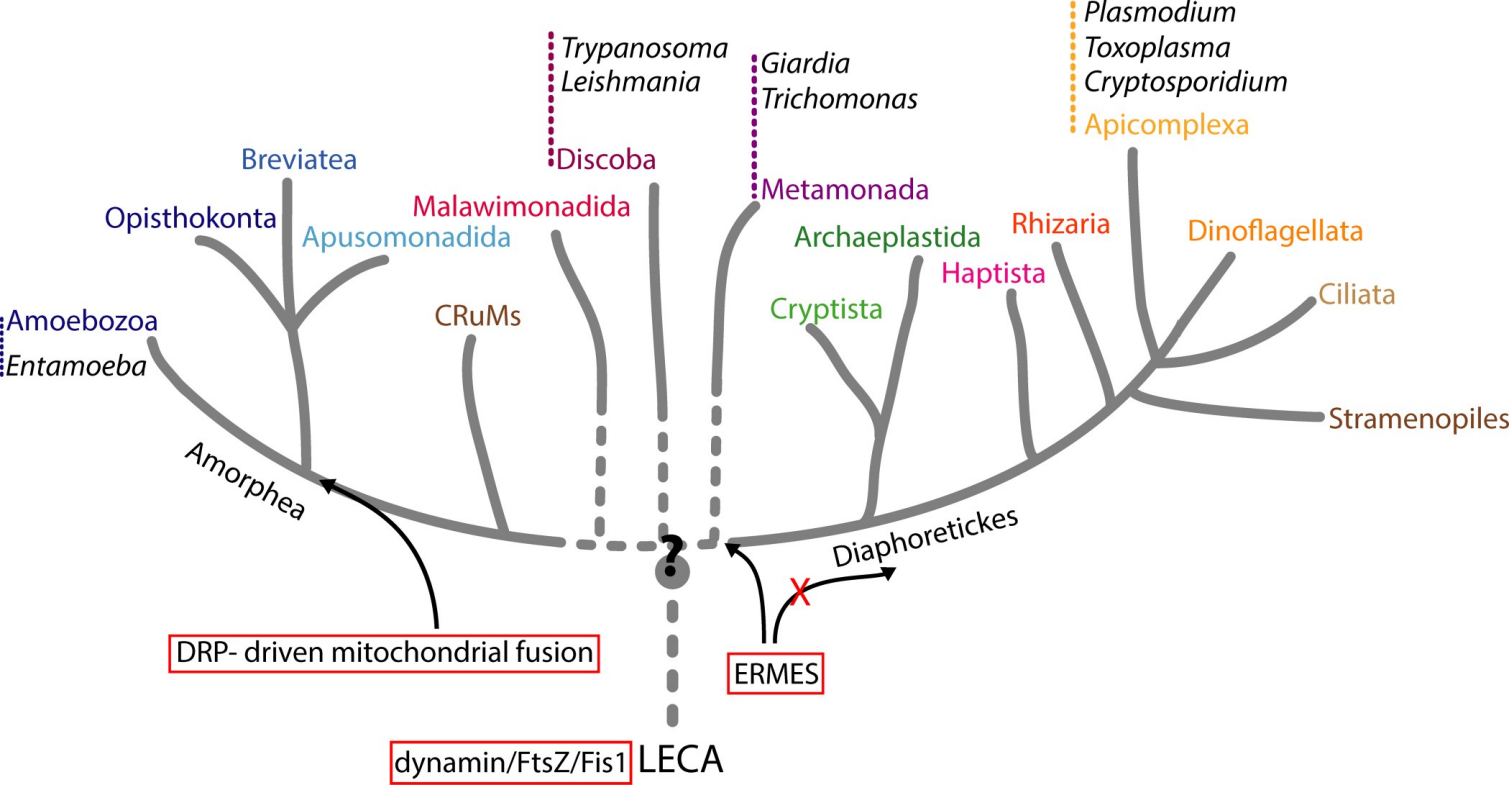
Parasitic protists in the eukaryotic tree of life. The schematic tree shows the position of the discussed protist parasites across the eukaryotic diversity. Currently, two large domains of eukaryotes called Amorphea and Diaphoretickes are distinguished with several additional clades of uncertain position [103]. *Plasmodium*, *Toxoplasma*, and *Cryptosporidium* belong to Apicomplexa, which together with Dinoflagellata and Ciliata constitute a group of Alveolata. Alveolata further combine with Stramenopiles and Rhizaria to form SAR group [103]. Discoba and Metamonada groups, which contain *Trypanosoma* with *Leishmania* and *Giardia* with *Trichomonas*, respectively, were previously part of the Excavata supergroup of eukaryotes. According to current classification, these groups belong neither to Amorphea or Diaphoretickes and remain without clear position in the tree (dotted lines). *Entamoeba* is part of the Amoebozoa supergroup. The comparative analyses proposed that the LECA already contained dynamin homologue, most likely capable of dual function in both mitochondrial division and vesicle scission [92]. There was also a mitochondrial targeted FtsZ, a prokaryotic homologue of tubulin, involved in the mitochondrial division [91]. Widespread distribution of Fis1 suggests that the protein was also used by LECA, although its specific involvement in mitochondrial division is unclear. Similarly, the components of the ERMES complex described as the molecular tether of the ER and mitochondria were common to Amorphea and the former Excavata group (Discoba, Metamonada, Malawimonada), while being absent in the common ancestor of Diaphoretickes [83]. However, the presence of individual ERMES components does not automatically imply the existence of functional ERMES complex. Mitochondrial fusion mediated by a DRP was confirmed only for the members of Amorphea and CRuMs; question mark depicts the uncertain position of the root of the tree. CRuMs, Collodictyonid + Rigifilida + *Mantamonas* clade; DRP, dynamin-related protein; ER, endoplasmic reticulum; ERMES, ER-mitochondria *e*ncounter *s*tructure; LECA, last eukaryotic common ancestor; SAR, Stramepiles-Alveolata-Rhizaria.

Obviously, there are multiple differences in mitochondrial morphology across the diversity of parasitic protists. Medically important parasitic protists belong to evolutionarily distinct supergroups of eukaryotes such as Stramepiles-Alveolata-Rhizaria (SAR; *Plasmodium*, *Toxoplasma*, *Cryptosporidium*), Discoba (*Trypanosoma*, *Leishmania*), Metamonada (*Giardia*, *Trichomonas*), and Amoebozoa (*Entamoeba*) (Fig 3). Our understanding of the mitochondrial dynamics comes mainly from the model organisms of the Opisthokonta group, which represent just a small piece of entire eukaryotic diversity (Fig 3). Interestingly, the comparative analyses have suggested that the mitochondrion of the last eukaryotic common ancestor (LECA) was already a complex organelle, with most of the biogenesis pathways resembling those in the current eukaryotes [90]. Its mitochondrion likely contained both the ancestral bacterial FtsZ-based machinery used for the bacterial cell division [91] and the dynamin-based membrane scission molecular machinery [92] perhaps accompanied by Fis1 receptor (Fig 3). Other complexes and proteins such as the ERMES complex or mitofusins arrived later in the evolution [83]. However, for most of the lineages of eukaryotes, we have only few functional data on the dynamics of mitochondria.

In the parasitic species, independent evolutionary adaptations have resulted in different demands on the metabolic pathways harbored in mitochondria. Consequently, the metabolic workload of mitochondria has been reflected by the shape and the size of the compartment. On top of that, mitochondria of protist parasites with digenetic life cycles dramatically change during the transition between hosts. Thus, many unrelated insect vector–borne parasites modulate their mitochondrial morphology according to their available energy source, i.e., the size of their mitochondria and the cristae within increases to break down amino acids by mitochondrial metabolism during invertebrate stages. Conversely, mitochondrial size is suppressed along with the mitochondrial metabolism in the glucose-rich environment of the vertebrate host. Anoxic environments occupied by some parasitic protists have led to the most extreme mitochondrial adaptations of miniature ATP-consuming organelles such as mitosomes. In fact, the discovery of a variety of mitochondria and MROs in the parasitic protists has fundamentally influenced our general understanding of the mitochondrial evolution [90].

However, remarkable similarities can be found among the mitochondrial dynamics of evolutionarily distant protist parasites. Their mitochondria do not undergo the extensive mitochondrial fusion and fission during the cell cycle as observed in Opisthokonta. Instead, in many species, mitochondrial fission is tightly connected to cell division and usually occurs just before cytokinesis. The lack of clear mitofusin homologues outside Opisthokonta (animals and fungi) (Table 1) indicates that the importance of mitochondrial fusion may be limited in protists and/or that different molecular machinery is in charge of fusing two organellar membranes. One of the main proposed roles of mitochondrial fusion is to control the quality of mitochondrial DNA among individual mitochondria in the cell, which may be damaged by reactive oxygen species generated during respiration [3]. Given that parasites from Apicomplexa and Kinetoplastida groups contain just a single mitochondrion with one mitochondrial nucleoid [51,93,94], this role of mitochondrial fusion may not be necessary. Conversely, MROs such as hydrogenosomes or mitosomes are usually present in large numbers in protists living in anoxic environments. However, these organelles are generally devoid of the organellar genome, which removes the necessity for quality control to ensure fidelity of nucleoid inheritance.

The constantly ongoing mitochondrial fusion and fission occurring in Opisthokonta allow the stochastic segregation of the mitochondria into daughter cells during the cellular division. It is possible that the synchrony of the mitochondrial and cellular division, observed for the protist parasites with single mitochondrion and also for the multiple mitosomes of *G*. *intestinalis*, might be a consequence of absent or reduced mitochondrial dynamics.

The role of mitophagy as an ultimate aspect of mitochondrial dynamics remains largely unexplored in parasitic protists, with the exception of *T*. *gondii* [45,46]. However, the concept of mitophagy of the only mitochondrion of *T*. *gondii* is somewhat complicated, as it should lead to cell death. Thus, further research is necessary to characterize the specific role of the mitophagy pathway in protist parasite biology.

Recent discovery of the ERMES complex, which mediates the connection of the mitochondria and the ER [13], showed that mitochondrial dynamics may also be controlled by other organelles. Further connections of the ERMES with the mitochondrial nucleoid replication and in mitochondrial contact site and cristae organizing system (MICOS) suggest that processes controlling mitochondrial dynamics are much more complex than previously thought [95,96].

Studies on these complexes in parasitic protists are now emerging [83,97], already indicating that their structure and function may be quite different from the model organisms, as usual for protists. [98–102].
